# Association Between Psychological Disorders and Irritable Bowel Syndrome

**DOI:** 10.7759/cureus.14513

**Published:** 2021-04-16

**Authors:** Salman Umrani, Waleed Jamshed, Amber Rizwan

**Affiliations:** 1 Internal Medicine, University Hospital of North Tees, Stockton-on-Tees, GBR; 2 Internal Medicine, Central Park Medical College, Lahore, PAK; 3 Family Medicine, Jinnah Post Graduate Medical Center, Karachi, PAK

**Keywords:** irritable bowel syndrome, anxiety, depression

## Abstract

Introduction

Irritable bowel syndrome (IBS) is an idiopathic, functional and chronic relapsing disorder. Physiological and psychological variables have been linked with etiology of IBS. In this study, we will determine the prevalence of IBS in local setting and its association with anxiety and depression.

Materials and methods

This cross-section study was conducted in multiple cities of Pakistan. One thousand and seven hundred and sixty (1,760) participants from general population between the age group 18 to 50 were enrolled in the study after informed consent. Diagnosis of IBS was made by assessing participants via ROME III criteria. Hospital anxiety and depression scale (HADS) was used to determine if the participants had anxiety and depression.

Results

IBS was present in 456 (25.9%) participants. IBS was significantly more prevalent in females compared to males. Anxiety was significantly more common in participants with IBS compared to participants without IBS (53.0% vs. 23.0%; p-value < 0.00001). Similarly, depression was significantly more common in participants with IBS (50.6% vs. 21.5%; p-value < 0.00001).

Conclusion

IBS is very common in Pakistan, but rarely diagnosed. It is important anyone, particularly at young age, presenting with diarrhea or constipation should be evaluated for IBS. Simultaneously, patients diagnosed with IBS should be screened for anxiety and depression, and managed accordingly.

## Introduction

Irritable bowel syndrome (IBS) affects 1 out of 10 persons of the general population worldwide [1]. IBS is an idiopathic, functional and chronic relapsing disorder, which may be caused due to psychosocial factors; altered motility, and/or altered sensory function of gastrointestinal tract [2]. IBS is characterized by abdominal pain and discomfort with frequent episodes of diarrhea and constipation [3]. It can be diagnosed clinically, based on exclusion of any organic disease [4]. Major causative agents that have been observed in occurrence of IBS includes prolonged infectious diseases, smoking, mucosal markers of inflammation, female gender, hypochondriasis and posttraumatic syndrome disease, etc. [5].

Physiological and psychological variables have been linked with etiology and severity of irritable bowel syndrome. Various studies have reported a high prevalence of psychiatric disorders in patients with IBS [6,7]. Globally lot of work has been done to establish association between IBS and psychological disorders. However, in developing countries, there is very limited data related to IBS and risk factors associated with it. In this study, we will determine the prevalence of IBS and its association with psychological disorders such as anxiety and depression.

## Materials and methods

This cross-section study was conducted in multiple cities of Pakistan. One thousand and seven hundred and sixty (1,760) participants from general population between the age group 18 to 50, of either gender, were enrolled in the study after informed consent. Participant’s age and gender were noted in self-structured questionnaire. Diagnosis of irritable bowel syndrome was made via ROME III criteria [8]. ROME III criteria define IBS as recurrent abdominal pain or discomfort for at least three days/months during the last three months associated with two or more of the following symptoms: improvement of pain with defecation, onset associated with a change in frequency of stool and onset associated with change in form of stool [8]. Patients diagnosed with IBS based on Rome III criteria were labelled as case group and patients negative for IBS was included as reference group.

To assess anxiety and depression among participants, Hospital anxiety and depression scale (HADS) was used. Score of less than eight (08) on each sub-scale of anxiety and depression indicated pathological anxiety and depression [9]. Interviewer filled the questionnaire after explaining each question to participant in the native language.

Statistical analysis was done using SPSS v. 24.0 (IBM Corporation, Armonk, NY, United States). Continuous variables were analyzed via descriptive statistics and were presented as means and standard deviations (SDs) while categorical data were presented as frequency and percentage. Chi-square was applied as appropriate. p-value of less than 0.05 meant that the difference between the groups is significant and the null hypothesis is void.

## Results

IBS was present in 456 (25.9%) participants. IBS was significantly more prevalent in females compared to males (61.7% vs. 38.3%; p-value < 0.00001). IBS was common in participants aged between 18 and 30 years (44.0%) (Table 1).

**Table 1:** Characteristics of case and control group. IBS; irritable bowel syndrome

Characteristics	IBS positive (n = 456)	IBS negative (n = 1,304)	p-value
Gender (%)			
Male	175 (38.3)	682 (52.3%)	<0.00001
Female	281 (61.7)	622 (47.7%)	
Age in years (%)			
18-30	201 (44.0%)	401 (30.7%)	<0.00001
31-40	155 (33.9%)	341 (26.1%)	
41-50	100 (21.9%)	562 (43.0%)	

In participants who were diagnosed with IBS based on ROME III criteria, the most common symptom was bloating (68.4%), followed by frequency of stool of more than 3 per day (44.0%) and abdominal pain relieved by passing stool (38.5%) (Table 2).

**Table 2:** Irritable bowel syndrome symptoms.

Irritable bowel syndrome symptoms	Frequency	Percentages
Bloating	312	68.4
Stool more than three per day	201	44.0
Abdominal Pain relieved by passing stool	176	38.5
Loose or watery stool	172	37.7
Stool less than three per week	152	33.3
Mucus in stool	62	13.5

Anxiety was significantly more common in participants with IBS compared to participants without IBS (53.0% vs. 23.0%; p-value < 0.00001). Similarly, depression was significantly more common in participants with IBS (50.6% vs. 21.5%; p-value, < 0.00001) (Table 3).

**Table 3:** Anxiety and depression in case and control group. HADS: hospitalized anxiety and depression scale.

HADS score	Anxiety			Depression		
	IBS positive (n = 456)	IBS negative (n = 1,304)	p-value	IBS positive (n = 456)	IBS negative (n = 1,304)	p-value
Less than 8	242 (53.0%)	301 (23.0%)	<0.00001	231 (50.6%)	281 (21.5%)	<0.00001
More than 8	214 (47.0%)	1003 (77.0%)		225 (49.4%)	1023 (78.5%)	

## Discussion

In this study, the prevalence of IBS was found to be more common in young people between the ages of 18-30 (44%). Majority of the participants with IBS in our study were reported to have symptoms of anxiety and depression. In our study, in patients who were diagnosed with IBS, the most common complaint was bloating (68.4%), followed by increase frequency of stool of more than three per day (44.0%) and abdominal pain relieved by passing stool (38.5%). American Gastroenterology Association (AGA) reported similar findings in a recent technical review [10]. It showed that psychological stress is known to aggravate gastrointestinal symptoms leading to severe diarrhoea, abdominal discomfort, etc. Moreover, psychological and psychiatric comorbidity is generally found among IBS patients. Women were reported to show supremacy over men in terms of having IBS, which is also believed to be present in women in the West [11]. 

Drossman et al. explored the association between IBS and psychological comorbidities, and found a positive link between the two. He found that 60% of patients with IBS have psychosocial problems [12]. Even though the exact origin of IBS is difficult to track down, it is believed that improper functioning of brain-gut pathways may be responsible for causing the disease [13]. This points towards the idea that abdominal symptoms have a tendency to affect anxiety and depression. These factors, in turn, affect the physiological factors [14]. Some other researches were able to prove the positive correlation between IBS and psychological comorbidities such as anxiety and depression [15-17]. However, there were some researches that could not find any such association [15,18-19].

A recent study by the Anxiety and Depression Association of America shown that seven out of 10 United States adults are reported to undergo stress and anxiety moderately every day [20]. Stress is an unavoidable and existing part of our life [21]. Recent studies have shown high prevalence of stress in university students [22,23]. Along with stress and anxiety, depression is also believed to be faced by a lot of college students. Wolfran et al. Stated in his study published in 2010 that in the USA, approximately 10% of university students are either diagnosed with depression, or were treated for it [24]. This correlates with findings of our study, which shows that the prevalence of IBS was more frequent in between age group of 18-30 years, which is a university-going age. 

Based on our study, we suggest that it is extremely important to provide psychiatric evaluation to anyone showing IBS-like symptoms, particularly in young population. As psychiatric symptoms can be presenting symptoms in patients with IBS, psychiatrists and psychologists should also be trained to screen for IBS, particularly in young population. Regular screening in universities and workplace for IBS may help in early diagnosis of IBS and may help alleviate the risk factors such as anxiety and stress, early in the course of disease.

To the best of our knowledge, it is the first study in local setting that studies the association of anxiety and depression with IBS. Considerably large sample size makes the finding of study reliable. Since data was collected from multiple cities, the sample was diverse. However, because of nature of study design, definite association cannot be established between IBS and psychological issues. Prospective studies are needed to consolidate the findings of our study.

## Conclusions

In this study, the prevalence of IBS was considerably high and prevalence of anxiety and depression in patients with IBS was also considerably high. It is important anyone presenting with gastrointestinal symptoms should be screened for diagnosis of IBS. After confirmation of IBS, patient should undergo complete psych evaluation for anxiety, stress and depression. Management of IBS should include management of anxiety, stress and depression as well.
